# Supplementary material for: Changes in Sedentary and Active Lifestyle, Diet Quality and Body Composition Nine Months after an Education Program in Polish Students Aged 11–12 Years: Report from the ABC of Healthy Eating Study
Source: Nutrients. 2019 Feb 3;11(2):331. doi: 10.3390/nu11020331 (PMC6412996; doi:10.3390/nu11020331)
Supplement: Supplementary file 1 [file nutrients-11-00331-s001.pdf]

nutrients

Changes in Sedentary and Active Lifestyle, Diet Quality and Body Composition Nine Months after an Education Program in Polish Students Aged 11–12 Years: Report from the ABC of Healthy Eating Study

Lidia Wadolowska, Jadwiga Hamulka, Joanna Kowalkowska, Natalia Ulewicz, Monika Hoffmann, Magdalena Gornicka, Monika Bronkowska, Teresa Leszczynska, Pawel Glibowski and Renata Korzeniowska-Ginter

Supplementary Materials

Table S1. Topics and details of the education program.

**Nutrition topic**	
Goal	Shaping pro-healthy dietary habits.
Scope	Nutrients important in the diet of young people. Health consequences of uncontrolled consumption of energy drinks and dietary supplements. A 'Good snack' as an alternative to chips, sticks and sweets. A Pyramid of Healthy Nutrition and Physical Activity and dietary guidelines for teenagers.
Methods	Talk; discussion; workshops.
Activities	Participants propose three various 'healthy' breakfasts to take at school by selecting foods from levels of the Pyramid of Healthy Nutrition and Physical Activity; Participants prepare an 'ideal sandwich'.
Tools	Brochure; puzzles; crosswords; website.
**Dietary topic**	
Goal	Supporting well-being, physical and intellectual development through a healthy lifestyle.
Scope	General recommendations for a healthy lifestyle (healthy eating and physical activity) at school age. The influence of nutrition and physical activity on physical and intellectual development and healthy well-being. The Student Menu—the brain cannot live only on chocolate—a discussion on the most important nutrients in the diet of young people.
Methods	Talk; discussion; workshops.
Activities	Using a pedometer to measure the number of calories consumed during the various activities. Determination of time needed to 'burn' the calories contained in the selected product. 'Nutrition detective'—measuring the amounts of oil, sugar and salt corresponding to fat, sucrose and salt content in various foods (three sets).
Tools	Brochure; puzzles; crosswords; website.
**Sensory–consumer topic**	
Goal	The world of senses. How to read food labels? What is important when choosing food?
Scope	Recognition of the basic flavours in aqueous solutions. Discovering the different taste sensations in selected foods. 'What kind of a consumer am I'—what do I like and why? Qualification of the consumer's personality.
Methods	Talk; discussion; workshops.
Activities	Use of sensory memory to identify eight coded odour samples of natural spices, vegetables and fruits. Recognition of selected foods with masked/closed eyes, based on sensory perceptions in the mouth. Preparation of colourful, tasty and healthy snacks from provided foods according to one's own ideas.
Tools	Brochure; recipes of 'healthy' snacks; website.
**Hygiene topic**	
Goal	Food safety. Hygiene during the preparation and consuming of meals.
Scope	The world of microorganisms, pathogens and probiotics. The rules of proper food storage and hygiene during meal preparation and consumption.
Methods	Talk; discussion; workshops.

Table S1. *Cont.*

Activities	Microscopic observation of selected microorganisms—Lactic acid bacteria (Lactobacillus) as an example of a microorganism with healthy properties, E. coli (Escherichia coli) as an example of a pathogen. Mapping the observed microorganisms' cells in the prepared templates. Practicing proper hand washing according to instructions. Checking hand cleanness with a test indicator, before and after hand washing.
Tools	Brochure; microscope; test indicator of hand washing; puzzles; crosswords; website.
**Culinary topic**	
Goal	How to prepare healthy, cheap and tasty meals?
Scope	The impact of culinary processes on sensory quality, nutritional value and food safety. The phenomenon of enzymatic browning of fruit and vegetables and ways to prevent this process.
Methods	Talk; discussion; workshops.
Activities	Culinary experiments—preventing the darkening of fruits and vegetables. Checking the impact of storage temperature on the quality of frozen foods, e.g., vegetables. Preparing low-budget healthy meals and low-sweetened beverages.
Tools	A 'healthy meals' recipe book; brochure; website.

nutrients MDPI

Table S2. Questions and correct answers (scored with 1 point) regarding an assessment of nutrition knowledge.

N	Question	Correct answer
1	The key to healthy eating is:	To eat many different kinds of foods and to eat more of some products than others and to eat moderate or small amounts of some products.
2	How much of milk and milk beverages, e.g., kefir, yoghurt, should you eat during a day?	Two glasses.
3	How much fruits and vegetables should you eat?	Five or more fruits and vegetables a day.
4	"Good" microorganisms are those microorganisms which:	Cause food fermentation and cause bread rising and have positive influence on human health.
5	Fast foods contain much of:	Fat and salt.
6	Which set of products contains much fibre?	Whole-meal bread, apple, bean.
7	Main sources of calcium in diet are:	Dairy products.
8	Which breakfast set contains less fat?	Corn flakes with full fat milk.
9	Main function of protein in the body is:	Regulation and structure.
10	Alkalizing products are:	Fruits, vegetables and potatoes.
11	Which information on the food label is most important for every consumer's food safety?	Shelf life.
12	Vegan diet means that one is eliminating from diet:	Meat, dairy and eggs.
13	Amount of human energy requirement depends on:	Age, sex and physical activity.
14	BMI is a ratio of:	Body weight and height.
15	To be active means:	Go to the gym and walk often and play sports e.g. football, volleyball, bike riding.
16	Which way of cooking is the best for vitamin C retention in potatoes?	Steam cooking.
17	Where one should keep an open container of juice:	In a refrigerator.
18	Are energy drinks safe for adolescents?	Should not be consumed before age of 16.

Table S3. Means and mean changes (95% CI) in nutrition knowledge, sedentary and active lifestyle, diet quality and body composition in total sample at a nine-month follow-up.

	**Baseline**	**Follow-up**	**Change: Follow-up − Baseline**
Nutrition knowledge score (points)	6.0 (5.7, 6.2)	7.9 (7.7, 8.2)	2.0**** (1.7, 2.3)
Screen time (points)	0.84 (0.74, 0.94)	0.97 (0.87, 1.06)	0.13 (0.04, 0.21)
Physical activity (points)	3.65 (3.53, 3.78)	3.51 (3.38, 3.63)	-0.15 (-0.25, -0.04)
pHDI (%)	27.7 (26.4, 29.0)	28.1 (26.8, 29.5)	0.6 (-0.8, 2.0)
nHDI (%)	14.3 (13.3, 15.3)	14.8 (13.8, 15.9)	0.5 (-0.5, 1.6)
z-WHtR (SDs)	0.01 (-0.09, 0.10)	-0.01 (-0.10, 0.08)	-0.02 (-0.08, 0.03)
z-BMI-for-age (SDs)	0.00 (-0.09, 0.09)	0.00 (-0.09, 0.09)	0.00 (-0.04, 0.04)
z-Waist circumference (SDs)	0.00 (-0.09, 0.09)	0.00 (-0.09, 0.09)	-0.01 (-0.06, 0.04)

Sample size may vary in variables due to missing data**.** Nutrition knowledge score (range: 0–18); Screen time (range: 0–5 points); Physical activity (range: 0–5 points); pHDI: pro-Healthy Diet Index (range: 0–100%); nHDI: non-Healthy Diet Index (range: 0–100%). Statistically significant (Mann–Whitney test): ****$p < 0.0001$.

nutrients MDPI

Table S4. Odds ratios (95% CIs) for nutrition knowledge, sedentary and active lifestyle, diet quality and body composition in educated and control groups at a nine-month follow-up.

	Baseline	Follow-up			
		Educated group		Control group	
		Crude model	Adjusted model	Crude model	Adjusted model
Nutrition knowledge score ≥Me (ref.: <Me)	ref.	3.61**** (2.49, 5.26)	3.78**** (2.57, 5.54)	3.38**** (2.06, 5.54)	3.51**** (2.10, 5.85)
Screen time ≥4 hours/day (ref.: <4 hours/day)	ref.	1.30 (0.87, 1.95)	1.31 (0.87, 1.97)	1.38 (0.81, 2.33)	1.39 (0.80, 2.40)
Adherence to WHO recommendation on physical activity (ref.: no adherence)	ref.	0.63** (0.45, 0.88)	0.61** (0.43, 0.86)	1.13 (0.68, 1.90)	1.12 (0.66, 1.89)
pHDI ≥Me (ref.: <Me)	ref.	1.06 (0.78, 1.43)	1.06 (0.77, 1.47)	1.00 (0.63, 1.59)	0.98 (0.61, 1.58)
nHDI ≥Me (ref.: <Me)	ref.	1.02 (0.75, 1.38)	1.05 (0.76, 1.44)	1.17 (0.73, 1.88)	1.17 (0.73, 1.87)
Central obesity (ref.: lack)	ref.	0.88 (0.55, 1.42)	0.83 (0.51, 1.36)	1.83 (0.59, 5.62)	1.63 (0.51, 5.22)
Thinness (ref.: normal)	ref.	1.04 (0.61, 1.76)	1.02 (0.59, 1.77)	0.70 (0.29, 1.67)	0.80 (0.31, 2.05)
Overweight/obesity (ref.: normal)	ref.	1.13 (0.78, 1.65)	1.09 (0.74, 1.61)	1.06 (0.64, 1.74)	0.98 (0.58, 1.67)
z-Waist circumference <−1 SD (ref.: −1 to 1 SD)	ref.	1.45 (0.81, 2.62)	1.28 (0.70, 2.37)	0.95 (0.55, 1.66)	0.93 (0.50, 1.71)
z-Waist circumference >1 SD (ref.: −1 to 1 SD)	ref.	1.12 (0.73, 1.71)	1.07 (0.69, 1.66)	1.47 (0.65, 3.35)	1.38 (0.56, 3.38)

Sample size may vary in variables due to missing data. Me: median; Median for Nutrition knowledge score: 6.0; Adherence to the WHO recommendations on physical activity (PA) was considered as meeting both vigorous PA at school (most of the time related to high physical exertion) and vigorous PA during leisure time (activities requiring physical effort over 3 hours/week)—details are given in Table 1; pHDI: pro-Healthy Diet Index; Median for pHDI: 25.875%; nHDI: non-Healthy Diet Index; Median for nHDI: 11.625%; Central obesity identified as a waist-to-height ratio of ≥0.5 according to Ashwell et al. [27]; Thinness, overweight/obesity and normal weight identified as BMI-for-age categorized with sex-specific cut-offs according to the International Obesity Task Force (IOTF) standards [28], as follows: thinness BMI < 18.5 kg/m^2; normal weight BMI = 18.5 to 24.9 kg/m^2; overweight/obesity BMI ≥ 25 kg/m^2; Odds ratios were adjusted for confounders (at baseline or follow-up, respectively). The adjustment for nutrition knowledge, screen time, adherence to WHO recommendation on physical activity, pHDI and nHDI was as follows: gender, age (years), residence (categorical variable), and Family Affluence Scale (points). The adjustment for central obesity, thinness, overweight/obesity, z-waist circumference <−1 SD and z-waist circumference >1 SD was as follows: confounders as mentioned above + screen time (points), physical activity (points), pro-Healthy Diet Index (%), and non-Healthy Diet Index (%). Statistically significant (Wald's statistics): **$p < 0.01$, ****$p < 0.0001$.
